# The importance of NOT being Other: Time to address the invisibility of nuanced gender and sexuality in clinical trials

**DOI:** 10.1186/s13063-023-07278-0

**Published:** 2023-03-30

**Authors:** Adam D. N. Williams, Kerenza Hood, Karen Bracken, Gillian W. Shorter

**Affiliations:** 1 Centre for Trials Research, Cardiff University, Cardiff, Wales, UK; 2 NHMRC Clinical Trials Centre, University of Sydney, Camperdown, NSW Australia; 3 Drug and Alcohol Research Network, Queen’s University Belfast, Belfast, Northern Ireland, UK

**Keywords:** Sex, Gender, Sexuality, Inclusivity, Trial recruitment, Trial data collection

## Abstract

**Background:**

Representation of all members of society within research, especially those typically underserved, is needed to ensure that trial evidence applies to the relevant population, and that effective interventions are available to all. The lack of appropriate and representative options in demographic questions around sex, gender and sexuality may result in the exclusion of LGBTQIA + people from health research.

**Main body:**

Sex and gender are not the same, yet this is rarely recognised in trial data collection, with the terms sex and gender often being used interchangeably. Sex or gender is often used as a stratification factor at randomisation and/or to define sub-groups at the time of data analysis, so correct data collection is essential for producing high-quality science. Sexuality also suffers from ‘othering’ with identities not being acknowledged but simply provided as an alternative to the perceived main identities. When collecting sexuality information, it is important to consider the purposes of collecting this data.

**Conclusion:**

We call on those involved in trials to consider how sex, gender and sexuality data are collected, with an active consideration of inclusivity. Through the description of all non-straight, non-cisgender people as ‘other’ you may be ignoring the needs of these populations and doing science, yourself, and them a disservice. Inclusivity may require small but important changes to ensure your research findings are inclusive and develop the evidence base for often overlooked populations.

## Background

With the development of the INCLUDE framework and the focus on COVID research, we have seen a growing attention to whether we provide equality of opportunity for involvement in research across our entire population and consideration as to if we are appropriately applying evidence-based care [1]. Many groups classified as underserved by research have considerably worse health outcomes and quality of life [2]. There are ways in which people are directly (such as through framing of trial inclusion/exclusion criteria) and indirectly ( through attitudes, language, processes, and procedures) excluded from trial participation. This is likely compounded by a lack of appropriate measurement, so we do not know if our research includes certain populations or not. This is particularly the case for people whose only option of response to some demographic questions is ‘other’, which obscures diversity in the population, whilst simultaneously presenting a lack of social engagement with the individual that may influence their consent to participate.

## Sex and gender are separate entities

One area of data collection and measurement in clinical trials that is very limited is sex and gender. Clinical trials lag substantially behind in this area with more inclusive sex and gender options broadly available in everything from government forms to online shopping. Sex is collected in most clinical trials but is often incorrectly used interchangeably with gender. Options for response are most often limited to binary options (male/female or man/woman). In regulatory reporting for pharmacovigilance to the MHRA, sex or gender are commonly reported in this way, although in regulatory terms the only area where this is required is around pregnancy (i.e. if the participant is pregnant or if a participant has got someone pregnant) [3]. Pregnant people as a group can diversely identify, and this could also be an opportunity for exclusion [4].

The key for any study is that they understand the population they have recruited from and represent that population in the data sample. This has an impact on the generalisability of the reported effect size, as well as the accuracy of the safety data. A more nuanced approach to considering sex and gender is needed.

One option for recording sex is to collect sex assigned at birth. However, this does not account for the impact that medical or surgical treatment may have had on the sex of transgender and gender-diverse participants, the impact of which may be clinically relevant to the trial. This may seem to all add complexity to what was a simple measurement of a binary classification or could be portrayed as political correctness but is necessary to ensure that the science is of high quality and that we understand safety across the total population affected. This variable is often used for stratification and sub-group analyses and so incorrect categorisation may affect estimates of effect, lowering the quality of findings. It is also worth noting that those who are a gender which differs from that they were classified as at birth have considerably worse health outcomes and quality of life [5] and are likely to be underserved in the INCLUDE Framework. For many trials, the multidimensional sex/gender measure may be sufficient and avoids ‘othering’ [6].

## Measuring sexuality

Sexuality is not in the standard data collection for all trials and is often only considered for studies which are about sexual or mental health. When it is collected, the available options may not be inclusive: often only considering if people are heterosexual or homosexual (straight or gay/lesbian) or ‘other’ with again a use of language that may exclude those who are of alternative sexualities (such as bisexual, pansexual, asexual). A challenge noted in survey literature but also potentially relevant in clinical trials is that this question may be challenging to answer with heterosexual people not recognising that as a label identifying them. The general grouping of sexual minorities (lesbian, gay men, bisexual, pansexual, asexual, etc.) into one homogenous group needs to be avoided as each sub-community will have its own needs and general patterns to health. For example, it is well documented that lesbians and bisexual women are substantially less likely to engage with preventive healthcare services and are more likely to be overweight or obese compared to heterosexual women [7], with gay and bisexual men having a higher burden of STIs and mental health problems but greater health literacy and engagement with sexual health services [8, 9]. Depending on the specifics of the clinical trial, it may be important to consider if these varying differences in health among populations will impact results and ensure that data is recorded correctly, acknowledging the varying identities of people whilst balancing this with trial efficiency (not collecting data that will not be analysed) and patient safety and privacy (right not to disclose). It is important not to assume who people are engaging in sexual contact with based on a measure of sexuality, straight identifying individuals are well documented to have same-sex sexual encounters and gay and lesbian individuals engaging in sex with the opposite sex.

## LGBTQ+ representation in research

The comments above highlight the problem that LGBTQ+ groups continue to face a lack of representation within general research, or often being omitted entirely. It is well documented that LGBTQ+ populations have poor physical and mental health outcomes, but the differences between groups are stark. Trans and all non-conforming gender groups have substantially worse physical and mental health outcomes compared to cisgender sexual minorities [9]. The negative health outcomes experienced among LGBTQ+ worsen in those from the additional underserved group. For example, LGBTQ+ youth are 2 to 3 times more likely to attempt suicide, with elderly LGBTQ+ individuals facing barriers to health due to isolation and poor cultural competence among social services. Both physical and mental health are significantly worse among LGBTQ+ ethnic minorities [8]. Highlighting the need for better consideration of intersectionality. The health situations of the people often grouped as ‘other’ do not adequately represent the complexity of health needs among these populations, with research needing to change its stance to accurately present these populations and improve their engagement.

## Conclusion

We now live in a world where most researchers will likely experience some form a representative data collection from the various sources that bombard us for information, yet accurate and diverse data collection continues to be omitted from clinical trials. What is holding us back? Is it ignorance (we hope not), complacency (only impacts what is incorrectly perceived to be a small proportion of people so is not a priority) or a concern that changing this will somehow damage our trials? As we recover from the pandemic and improve the ways we work now is the time to change trial methodology for the better and create a more inclusive way of conducting research. For all those involved in clinical trials, we ask you to consider how you collect sex, gender and sexuality data in your trials and is your data collection being inclusive? Even if your data cannot be analysed through these groups, advances in data synthesis such as individual patient data meta-analysis, can better understand their needs through data synthesis [10]. Through the description of all non-straight, non-cisgender people as ‘other’ are you ignoring the needs of these populations and doing science, yourself, and them a disservice.

## Data Availability

Not applicable.
